# Working memory processes and the histamine-3 receptor in schizophrenia: a [^11^C]MK-8278 PET-fMRI study

**DOI:** 10.1007/s00213-024-06730-6

**Published:** 2024-12-22

**Authors:** Atheeshaan Arumuham, Ekaterina Shatalina, Matthew M. Nour, Mattia Veronese, Ellis Chika Onwordi, Stephen J. Kaar, Sameer Jauhar, Eugenii A. Rabiner, Oliver D. Howes

**Affiliations:** 1 Department of Psychosis Studies, Institute of Psychiatry, Psychology & Neuroscience, Kings College London, De Crespigny Park, London, SE5 8AF UK; 2 Institute of Clinical Sciences (ICS), Faculty of Medicine, Imperial College London, London, W12 0NN UK; 3 Psychiatric Imaging Group, Medical Research Council, London Institute of Medical Sciences, Hammersmith Hospital, London, W12 0NN UK; 4 Department of Psychiatry, University of Oxford, Oxford, OX3 7JX UK; 5 Max Planck University College London Centre for Computational Psychiatry and Ageing research, London, WC1B 5EH UK; 6 Department of Information Engineering, University of Padua, Padua, Italy; 7 Department of Neuroimaging, Institute of Psychiatry, Psychology and Neuroscience, Kings College London, London, UK; 8 Centre for Psychiatry and Mental Health, Wolfson Institute of Population Health, Queen Mary University of London, London, E1 2AB UK; 9 Division of Psychology and Mental Health, Faculty of Biology, Medicine, and Health, The University of Manchester, Manchester, M13 9WL England; 10 Greater Manchester Mental Health NHS Foundation Trust, Addictions Services, Manchester, M25 3BL England; 11 Psychological Medicine, Institute of Psychiatry, Psychology and Neuroscience, Kings College, London, UK; 12 Invicro, London, W12 0NN UK; 13 H Lundbeck A/s, 3 Abbey View, Everard Close, St Albans, AL1 2PS UK

**Keywords:** Schizophrenia, Histamine, H3R, Cognitive impairment, Working memory, Neuroimaging, PET, FMRI

## Abstract

**Rationale:**

Working memory impairment is a prominent feature of schizophrenia which predicts clinical and functional outcomes. Preclinical data suggest histamine-3 receptor (H3R) expression in cortical pyramidal neurons may have a role in working memory, and post-mortem data has found disruptions of H3R expression in schizophrenia.

**Objectives:**

We examined the role of H3R in vivo to elucidate its role on working memory impairment in schizophrenia.

**Methods:**

We used positron emission tomography (PET) with the selective H3R radioligand [^11^C]MK-8278 to measure H3R availability, and employed a task during functional magnetic resonance imaging (fMRI) to assess working memory-evoked brain activation and cognitive task performance, in patients with schizophrenia (*n* = 12) and matched healthy volunteers (*n* = 12). We assessed the relationship between H3R availability and both task performance and working memory-evoked brain activation in regions of interest (ROIs), including the anterior cingulate cortex (ACC) and dorsolateral prefrontal cortex (DLPFC).

**Results:**

Patients with schizophrenia showed a strong positive correlation, after corrections for multiple comparisons, between ACC H3R availability and task performance (rho = 0.73, *p* = 0.007), which was absent in the control group (rho = 0.03, *p* = 0.94). Further ROI analysis did not find a significant relationship between H3R availability and working memory-evoked brain activation.

**Conclusions:**

These results provide support for the role of H3R on working memory processes in patients with schizophrenia.

**Supplementary Information:**

The online version contains supplementary material available at 10.1007/s00213-024-06730-6.

## Introduction

Schizophrenia is a chronic and debilitating neuropsychiatric condition which has a profound impact on the quality of life of patients and carers (Caqueo-Urízar et al. 2009; Dong et al. 2019). Along with positive (e.g., delusions, hallucinations, disorganised behaviour) and negative symptoms (e.g., anhedonia, asociality, blunted affect), cognitive impairment associated with schizophrenia (CIAS) is a common feature of the condition, with onset of cognitive impairment often preceding positive symptoms (Cornblatt and Erlenmeyer-Kimling 1985; Davidson et al. 1999; Bora and Murray 2013; Jauhar et al. 2022) and persisting the course of illness (Green 2006; Zanelli et al. 2019). Notably, more severe CIAS is associated with greater functional disability (Bowie and Harvey 2006).

Several cognitive domains are negatively affected in schizophrenia including: speed of processing, attention, verbal learning and memory, visual learning and memory, executive functioning, verbal comprehension, social cognition, and working memory (Nuechterlein et al. 2004). Impairments in working memory are among the most marked components of CIAS (Wilk et al. 2005; Reichenberg et al. 2009). Working memory is a finite and temporary short term memory store, which involves perceptual attention for encoding, active maintenance, and manipulation of novel informative stimuli in a readily accessible form (Klink et al. 2017). In schizophrenia, greater severity of working memory impairment is predictive of positive psychosis symptom severity (Jenkins et al. 2018; Panov et al. 2023) and functional outcomes (Cervellione et al. 2007; Shamsi et al. 2011). In humans and non-human primates, the cingulo-prefrontal network has been implicated as having a major role in working memory function, with increased engagement of these regions during working memory processes and increased activation of excitatory pyramidal neurones (Owen et al. 2005; Chein et al. 2011; Medalla and Barbas 2012). Functional magnetic resonance imaging (fMRI) studies have provided evidence of dysfunctional activation of the anterior cingulate cortex (ACC) and dorsolateral prefrontal cortex (DLPFC) regions in patients with schizophrenia compared to healthy volunteers when undergoing working memory tasks (Glahn et al. 2005; Minzenberg et al. 2009; Wu and Jiang 2020; Wang et al. 2021). Such dysfunction might reflect imbalance of excitatory and inhibitory dynamics within cortical circuits, disrupting an ability of such circuits to maintain working memory representations in absence of stimuli (Murray et al. 2014). However, the molecular mechanisms underlying the brain activation alterations and impairment in working memory remain unclear. Understanding this could identify targets for the development of novel treatments for CIAS (Kaar et al. 2020; McCutcheon et al. 2023).

The histamine 3 receptor (H3R) is a presynaptic receptor which, when activated, inhibits the release of excitatory and inhibitory neurotransmitters in cortical neurones that are implicated with cognitive function including working memory, such as glutamate, acetylcholine, noradrenaline, dopamine and gamma-aminobutyric acid (Arrang et al. 1987; Schlicker et al. 1988, 1989, 1993; Clapham and Kilpatrick 1992; Morales-Figueroa et al. 2019). The glutamatergic corticostriatal pathway has an essential role in working memory function (Haber 2016). H3R has high expression on these neurones where they have been found to influence the development of corticostriatal circuits (Han et al. 2020). Receptor activation depresses striatal synaptic transmission and modulates excitatory and inhibitory signalling (Doreulee et al. 2001; Ellender et al. 2011). Of note, in animal models of epilepsy, a condition which features an imbalance of excitatory and inhibitory signalling (Duma et al. 2024), H3R antagonists have been found to have effective anticonvulsive properties (Murakami et al. 1995; Ishizawa et al. 2000; Vohora et al. 2000; Chen et al. 2002). This effect is attributed to the ability of H3R to control the release of GABA and glutamate (Bhowmik et al. 2012). Therefore, dysfunction of H3R signalling may plausibly affect the excitation-inhibition signalling balance observed in schizophrenia, the disruption of which has also been associated with working memory impairment (Gonzalez-Burgos et al. 2015; Hoftman et al. 2017, 2018; Calvin and Redish 2021). 

Mice lacking H3R have been found to have enhanced performance in cognitive tasks that employ working memory (Toyota et al. 2002; Rizk et al. 2004). Moreover, H3R antagonist studies in rodents have found these compounds attenuate impaired alternation performance in the cross-maze task, which is used as an indication of working memory, in animal models of cognitive impairment (Brown et al. 2013). Similarly, H3R antagonists have been found to improve performance in other tasks in animal studies such as passive avoidance, water maze learning, object recognition, and odour discrimination; tasks associated to learning and memory consolidation (Medhurst et al. 2007; Foley et al. 2009). Additionally, H3R antagonists improve prepulse inhibition (PPI) deficits in rodents (Fox et al. 2005; Raddatz et al. 2012), which is a well-replicated feature of schizophrenia and is attributed to sensorimotor gating dysfunction (Graham 1975; Hoffman and Ison 1980; Kumari et al. 2000). Importantly, sensorimotor gating functions, which are involved with the perception of information, also strongly contribute to the short-term retention of such information that is required for working memory (D’Esposito and Postle 2014; Oliveras et al. 2015).

Related to this, a multi-modal PET-fMRI study of healthy volunteers reported a negative relationship between DLPFC H3R distribution volume (V_T_) and working memory-related blood oxygen level dependent (BOLD) activation in the DLPFC (Ito et al. 2018). These lines of evidence suggest higher cortical H3R availability in schizophrenia could underlie the altered activation that is seen in these regions during working memory tasks, with the molecular basis potentially owing to disruption of the excitation-inhibition balance. This has not previously been examined in patients with schizophrenia.

To address this, we aimed to investigate the relationship between working memory and H3R availability in both patients with schizophrenia and healthy volunteers. We hypothesised that both patients with schizophrenia and healthy volunteers would show an inverse association between H3R availability and working memory-related BOLD activation in the ACC and DLPFC. We also hypothesised that higher H3R availability in the ACC and DLPFC would be associated with poorer performance in the cognitive task employed.

## Methods

### Ethics statement

The study was approved by the West London & GTAC Research Ethics Committee (REC reference: 17/LO/1299) and the Administration of Radioactive Substances Advisory Committee (ARSAC licence: 630/3764/36826). All participants provided written informed consent to participate, following confirmation from a clinician of their capacity to do so. We followed the Strengthening the Reporting of Observational Studies in Epidemiology (STROBE) reporting guidelines for case-control studies. The study was conducted in accordance with the Declaration of Helsinki.

### Participants

The study recruited two groups, consisting of patients with schizophrenia (*n* = 12; male = 9, female = 3, mean age [SD] = 30.4[13.0]) and matched healthy volunteers (*n* = 12; male = 9, female = 3, mean age [SD] = 30.3[11.5]). Patients were recruited from community mental health teams in the South London and Maudsley NHS Foundation Trust, United Kingdom. Healthy volunteers were recruited via approved advertisements that were locally distributed. Power calculations to determine sample size were performed using the G*Power 3.1 software (ver. 3.1.9.7; Heinrich-Heine-Universität Düsseldorf, Düsseldorf, Germany) (Faul et al. 2007, 2009). Based on a study previously reporting the association of working memory load during the n-back task and H3R availability in the DLPFC (Ito et al. 2018), a power calculation indicated a total sample size (including healthy volunteers and patients) of 24 would have > 95% power to detect a relationship of *R*^*2*^ = 0.42, *p* < 0.05 (two-tailed), between working memory load and H3R availability in the DLPFC. No previous study has explored such a relationship in the ACC. In view of this, we used the prior evidence in the DLPFC to hypothesise a similar strength relationship in the ACC as well.

In total *n* = 30 participants were recruited for the study, including *n* = 16 healthy volunteers and *n* = 14 patients. A total of *n* = 3 participants (*n* = 2 healthy volunteers, *n* = 1 patient) withdrew consent prior to scanning. Of the remaining participants, *n* = 3 (*n* = 2 healthy volunteers, *n* = 1 patient) withdrew consent following the MRI scan. A total of *n* = 24 participants (*n* = 12 healthy volunteers, *n* = 12 patients) received both a PET and MRI scan. Data were collected from August 16th, 2018, to March 24th, 2021. The PET but not fMRI data have been previously published (Arumuham et al. 2023).

### Inclusion and exclusion criteria

Inclusion criteria were as follows: for the patient group a diagnosis of schizophrenia, as determined by a psychiatrist, according to the *Structured Clinical Interview of DSM-IV-TR Axis I Disorders-Patient Edition* (First et al. 2002), for the healthy control group, that were matched for both sex and age (+/- 3 years), no current or lifetime history of an Axis I disorder as determined by the *Structural Clinical Interview of DSM-IV-TR Axis I Disorders-Patient Edition* (First et al. 2002).

Exclusion criteria for all particpants were as follows: history of significant head trauma (resulting in loss of consciousness > 1 minute or requiring hospital admission), negative Allen’s test indicating inadequate collateral circulation of the hand, dependence on illicit substances or alcohol, positive urine drug test (SureScreen Diagnostics, Derby, UK) for any illicit substances that might affect H3R (e.g. stimulants) on the day of scanning, medical comorbidity (other than minor illnesses), current use (within the last 3 months) of H3R modulating medications (e.g., pitolisant), and contraindications to scanning (such as claustrophobia and pregnancy). Clozapine was the only antipsychotic that was not permissable during this study as it has comparatively higher affinity for H3R compared to other antipsychotic drugs, although it is still thought to have a weak affinity overall (Rodrigues et al. 1995; Appl et al. 2012; Humbert-Claude et al. 2012) (see the Supplementary Material for full inclusion and exclusion criteria).

### Clinical and demographic measures

Clinical symptom severity in the patient group was assessed using the Positive and Negative Syndrome Scale (PANSS) (Kay et al. 1987). Psychotropic medication history was recorded, urine drug screens were performed, and dose of antipsychotic treatment was converted to chlorpromazine equivalent dose using previously reported methods (Leucht et al. 2014). Demographic data collected included sex, age, substance use, medical and drug history.

### Neuroimaging

#### PET acquisition

Scans were performed on a Siemens BioGraph 6 HiRez PET-CT scanner (Siemens, Erlangen, Germany). All participants underwent a dynamic, continuous 90-minute PET acquisition after a bolus injection of [^11^C]MK-8278 which is a radiotracer with high affinity and selectivity for H3R (Van Laere et al. 2014). To control for diurnal variation of histamine release (Mochizuki et al. 1992; Brown et al. 2001; Burns et al. 2003), all scans were performed during the same time period (1000–1300). A low-dose CT topogram (0.36 mSv) was acquired prior to PET acquisition for attenuation correction during the PET image reconstruction. PET emission data were corrected for attenuation and scatter, then reconstructed using Fourier re-binning and 2D filtered back projection with a 2.0 mm kernal Ramp filter, into 26 frames according to the following binning: (8 × 15s,3 × 60s,5 × 120s,5 × 300s,5 × 600s). The final reconstructed data had voxel dimensions of 2.051 × 2.051 × 2.000 mm^3^. In parallel to PET imaging, continuous arterial sampling using a blood sampler (Allogg ABSS (Allogg AB, Mariefred, Sweden, http://www.allogg.se/) was performed for the first 15 min followed by 12 discrete samples to measure radiotracer levels in blood (for the full acquisition protocol please see Supplementary Material).

#### PET analysis

H3R availability was determined as the [^11^C]MK-8278 volume of distribution (V_T_, mL/cm^3^), calculated using the standard 2-tissue compartmental modelling (2TCM) method that has superior fitting performance compared to 1-tissue compartmental modelling (1TCM) (Arumuham et al. 2023), with a metabolite-corrected arterial plasma input function (see Supplementary Material for further information on PET image analysis and model validation). The ACC and DLPFC were selected as primary regions of interest (ROIs) because of the the influence of H3R on cognition by modulating neurotransmitter release in these regions, along with having an established role in working memory which is disrupted schizophrenia (Glahn et al. 2005; Mahmood 2016). The Clinical Imaging Centre (CIC) atlas was used to define ROIs described above, as previously described (Tziortzi et al. 2011). Using Statistical Parametric Mapping 12 (SPM12; version 6684)(The FIL Methods Group 2014), grey matter (GM) masks were obtained by binarising segmented GM from T1-weighted images and applying this to the CIC atlas.

#### MRI acquisition

Prior to fMRI data acquisition, all subjects underwent structural imaging to optimise anatomical delineation of ROIs. T1-weighted three-dimension magnetisation-prepared rapid acquisition gradient echo (MPRAGE) images were acquired on a Siemens Magnetom Verio Syngo MR B17 3T scanner (Siemens, Erlangen, Germany), using a 32-channel phased-array head-coil, according to the following parameters: repetition time = 2300.0 ms, echo time = 2.98 ms, flip angle = 9°, field of view (FOV) = 256 × 256 mm, 160 sagittal slices of 1-mm thickness, distance factor = 50%, voxel size = 1.0 × 1.0 × 1.0 mm.

Functional data were acquired over 9 min, during which time participants completed the n-back task detailed below. Whole-brain functional echo-planar images (EPI) were acquired with 270 whole-brain volumes per session, consisting of 72 interleaved slices (2 mm thickness), with a repetition time = 2000 ms, echo time = 30 ms, in-plane resolution = 3 × 3 mm, flip angle = 62°, and bandwidth = 1906 Hz/pixel.

#### fMRI n-back task

**Fig. 1:**
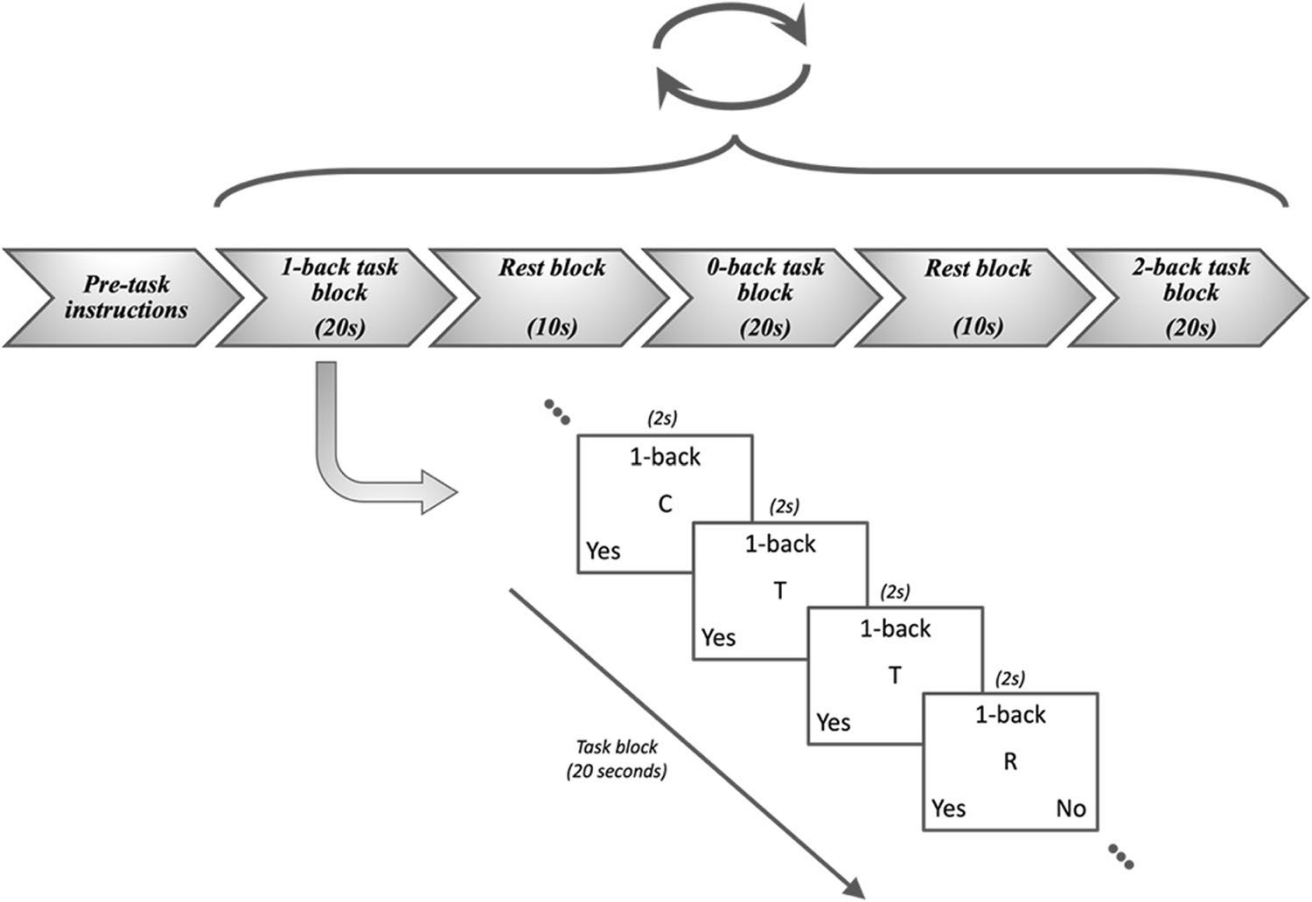
Design of the letter n-back task comprised of 0-, 1-, & 2-back task blocks each lasting 20s, with each trial letter being shown on the screen for 2s each. Participants were asked to respond either “yes” or “no” as quickly as possible, via buttons on an MR-compatible box according to the task condition. Task blocks are separated by rest blocks lasting 10s. Upon completing a cycle of 0-back, 1-back, & 2-back tasks, these three blocks were pseudo-randomised and readministered to participants, for a total of 6 cycles (made up of 18 task blocks with an equal number of 0-, 1-, & 2-back conditions)

Participants completed a block-design, n-back task during fMRI scanning, known to engage working memory, and adapted from a previous study (Nour et al. 2019). This task was coded in PsychoPy version 2 (https://www.psychopy.org). Participants completed 18 task blocks of a letter n-back task, made up of 6 blocks each of 0-, 1-, and 2-back conditions (see Fig. 1). During the 0-back block, participants were required to remember an initial target letter, and identify whether the subsequent letters within a sequence matched the target letter. During the 1- and 2-back blocks, the participants had to recall whether the subsequent letters within a sequence matched the letter presented 1 or 2 trials prior, respectively. Participants were asked to respond as quickly as possible, using an MRI-compatible response box, with the index and middle fingers of their dominant hand (indicating ‘yes’ and ‘no’ respectively). Each block lasted 20 seconds in total, containing 10 two-second trials, and was followed by a 10-second rest period. The 18 task blocks were pseudo-randomised to 0-, 1-, 2-back conditions, with 6 blocks of each condition per task session. Reaction times and accuracy (as a percentage of correct responses) were recorded for each task condition. As per the previous study assessing H3R availability and working memory in healthy volunteers, we used task accuracy as a marker for task performance (Ito et al. 2018). No subjects were excluded from analysis due to poor performance in the task, defined as accuracy less than 50%.

#### fMRI analysis

All functional and anatomical data were pre-processed with FSL (FMRIB Software Library v5.0.4; http://www.fmrib.ox.ac.uk/fsl/). The Brain Extraction Tool (BET) was used for brain extraction of the anatomical data while the fsl_anat script was used for anatomical data preprocessing. Motion correction was performed with FMRIB Linear Image Registration Tool (MCFLIRT), with smoothing performed using a Gaussian kernel of full width at half maximum (FWHM) of 6 mm. Temporal high-pass filtering was applied with a 100s cut-off threshold. Firstly, for co-registration, functional data was registered to the subject’s individual anatomical image and then to an anatomical template image in standard stereotactic space (MNI152). Data were excluded if the maximum motion for a subject exceeded one voxel dimension (3 mm) across multiple volumes. No data were excluded due to excess motion.

Voxel-wise first-level (single-subject) analyses were carried out in FSL’s FEAT module using the general linear model (GLM) and FILM (FMRIB’s Improved Linear Model) pre-whitening. The haemodynamic response function (HRF) was modelled with 0-back, 1-back, and 2-back conditions as explanatory variables, modelled with boxcar regressors. Head motion regressors were included in the model. Task regressors were convoluted with a standard Gamma function (SD = 3s, Mean lag = 6s). Temporal derivative and filtering to match the pre-processing steps were applied to the data (Aguirre et al. 1998). For each participant, we operationalised working memory-related activation as a statistical of BOLD responses for the 1- & 2-back > 0-back condition, to assess working memory-load.

We then conducted a whole-brain second-level (group) analysis that compared this mean working memory-load contrast between patients with schizophrenia and healthy volunteers at each voxel, using two-sample t-tests, implemented in FSL’s FLAME-1 model. For group-level statistical inference, we used a whole-brain cluster-corrected significance threshold (cluster defining threshold of Z = 2.3, whole-brain family-wise error corrected *p* < 0.05) (Eklund et al. 2016).

#### PET - fMRI analysis

For multimodal analyses, we extracted H3R availability (PET) and working memory-related neural response (fMRI) from identical anatomical regions of interest (ROIs). As stated, these were chosen due to evidence of H3R affecting working memory function through modulating neurotransmitter release, along with meta-analytic data of disrupted working memory-task evoked activation in schizophrenia within the selected ROIs (Glahn et al. 2005; Mahmood 2016). ROIs, defined using the CIC atlas during PET analysis, were transformed into masks (see Supplemental Material). Thus providing anatomically identical ROIs for both PET and fMRI analysis. FSL’s featquery module was used to transform these masks into individual subject space. BOLD response, measured as the mean parameter estimates (beta values) across all voxels in each ROI, were extracted for each subject from their first-level analyses.

### Statistical analysis

Statistical Product and Service Solutions (SPSS) version 22 (IBM Corp) was used for all statistical analyses. The significance level was set at *p* < 0.05 (two-tailed, corrected). The Shapiro-Wilk test was used to assess for normality of distribution, and ouliers were detected using the Tukey method (Tukey 1977). Categorical clinical, demographics, and experimental variables were compared across groups using χ^2^ tests. Continuous variables were assessed using independent-samples t-tests for parametric data, while Mann-Whitney tests were used for non-parametric data.

Analysis of variance (ANOVA) was performed to examine tracer uptake, which assessed for both the main effect of group status, and diagnosis x ROI interaction. Where appropriate, post-hoc pairwise independent samples t-tests were performed. For group analysis of BOLD response, mean ROI parameter estimate values for the 1- & 2-back > 0-back condition were compared using independent samples t-tests. Outliers were tested for and excluded from further analysis, using the Tukey method within SPSS (Tukey 1977).

To determine the relationship between H3R availability, measured as [^11^C]MK-8278 V_T_, and working memory-related BOLD response, measured as the mean parameter estimates across all voxels in each ROI in the 1- & 2-back > 0-back contrast, we conducted Pearson’s correlation. Similarly, Pearson’s or Spearman’s correlation were used to assess the relationship between H3R availability and n-back task performance, for normal and non-normally distributed data respectively. Post-hoc comparison of correlation coefficients between patients and controls were performed using a z-test on Fisher r-to-z transformed correlation coefficients, which directly compares correlation coefficients (Hinkle et al. 1988). Independent samples t-tests were also performed to carry out ROI volumetric analysis in order to determine the potential impact of regional volume difference on findings.

## Results

### Demographics and experimental variables

There were no significant differences between groups for age (Mann-Whitney U = 67.5, *p* = 0.80), or weight (t_22_= −1.26, *p* = 0.22), and groups were matched for sex (see Table 1). On average, all participants received an average [^11^C]MK-8278 injected dose of (mean ± SD), 263.59 ± 18.67 MBq, while patients received 9% less injected dose than controls. Injected dose showed no significant correlation with V_T_ values (ACC: Spearman’s rho = 0.04, *p* = 0.84; DLPFC: rho = 0.16, *p* = 0.49), and thus was not corrected for in further analysis.

**Table 1:** Demographics and experimental variables. Abbreviations: shaded box, not applicable; PANSS, positive and negative syndrome scale; SD = standard deviation; n = number, *= 𝛘² test, ^= Mann-Whitney U test, #= independent sample t-test

	Healthy volunteers (*n* = 12)	Patients (*n* = 12)	Effect size	*p*
Sex, male : female	9 : 3	9 : 3	φ = 0	1.00 *
Age, years, mean (SD)	30.3 (11.5)	30.4 (13.0)	*Cohen’s d* = 0.11	0.80 ^^^
Weight, kg, mean (SD)	73.1 (12.4)	79.7 (13.4)	*Cohen’s d* = 0.51	0.22 ^#^
Injected dose, MBq, mean (SD)	275.6 (10.7)	251.5 (18.1)	*Cohen’s d = 2.07*	< 0.01 ^^^
Illness duration (years)		5.8 (6.3)		
Antipsychotic free, n (%)		7 (58.3%)		
Chlorpromazine equivalent dose/ mg, mean (SD)		324.3 (118.2)		
PANSS positive, mean (SD)		16.8 (3.7)		
PANSS negative, mean (SD)		17.7 (3.9)		
PANSS general, mean (SD)		35.4 (7.5)		
PANSS total, mean (SD)		69.8 (12.7)		

### Task performance data

**Fig. 2:**
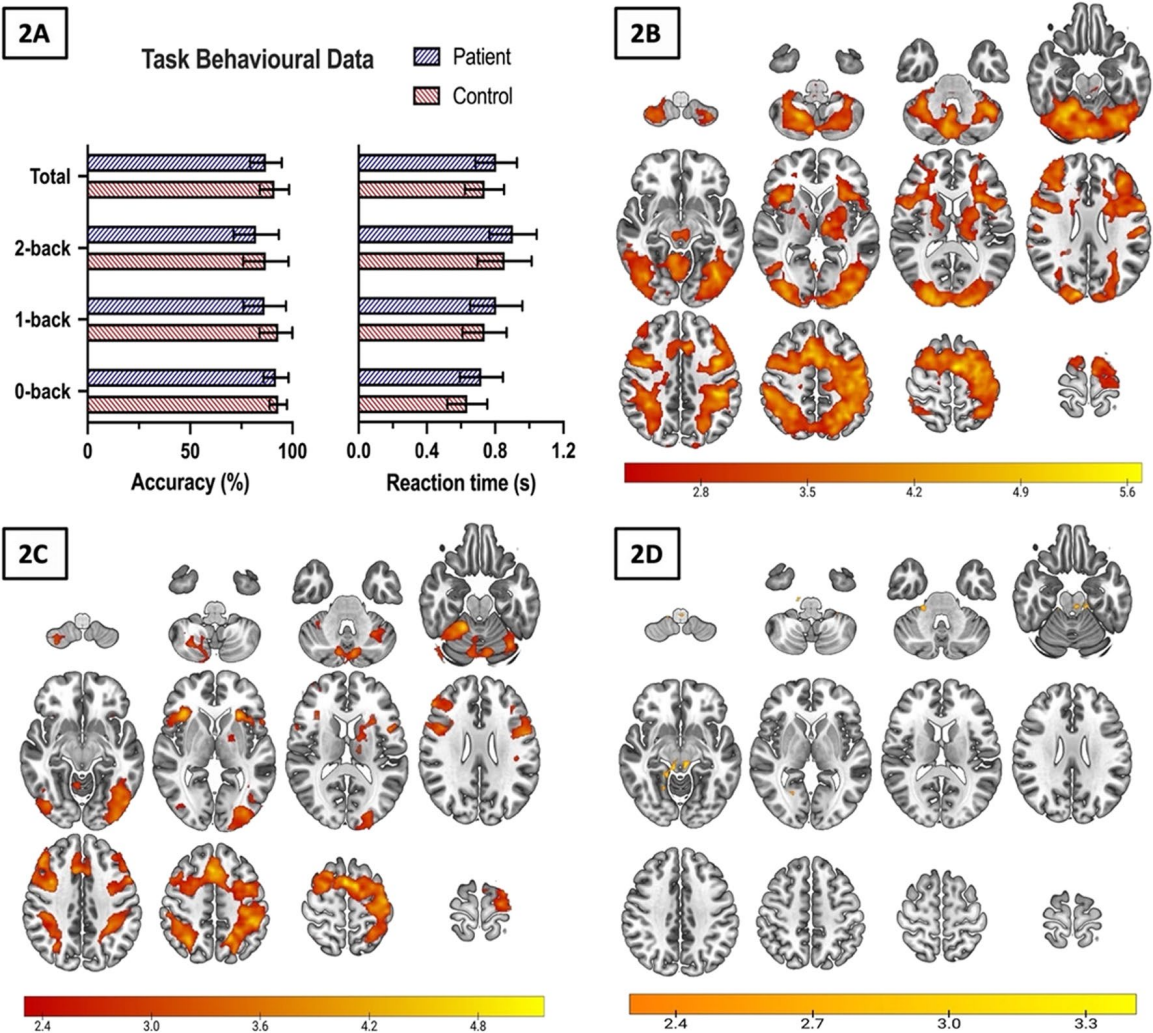
Task behavioural data and working memory-related BOLD activation. Figure 2**A** portrays beharioural data for both groups during the n-back task, including both accuracy (recorded as a percentage) and reaction time (recorded in seconds). Individual task condition data (i.e., 0-, 1-, & 2-back) are shown, as well as a total score across the whole task. Data shown are mean, with error bars indicating standard deviation. 2**B **and 2**C** represent mean working memory-related activation in control and patient groups respectively. 2**D** displays significantly higher activated clusters in controls compared to patients with schizophrenia for the 1- & 2-back > 0-back contrast. All imaging data indicates mean activation during the n-back task applying the contast condition of 1- & 2-back > 0-back. Z (Gaussianised T/F) statistical images were thresholded at the whole-brain level using clusters determined by z > 2.3 and a corrected cluster significance threshold of *p* = 0.05. Colour bars indicate Z-scores for corresponding imaging data

N-back performance was measured as total task accuracy. As a sensitivity analysis we also collected reaction speed data. We found no significant difference between groups for either total task accuracy (control mean ± SD = 91.2 ± 0.7%, patient mean ± SD = 87.1 ± 0.08%, t_22_ = 1.35, *p* = 0.19) or total reaction time (control = 0.74 ± 0.11 s, patient = 0.81 ± 0.12 s, t_22_ = −1.41, *p* = 0.17). Moreover, we did not find a significant difference in performance, when comparing specific task conditions between groups (i.e., 0-back, 1-back, and 2-back) (see Fig. 2A). When assessing difference in accuracy across the three n-back tasks within groups (i.e., 0-back v 1-back v 2-back), we found a consistent significant effect of load on performance measures in both controls (accuracy: *χ*^2^ = 10.5, *p* = 0.005; reaction time: *χ*^2^ = 20.7, *p* < 0.001, df = 2; Friedman Test) and patients (accuracy: *χ*^2^ = 9.0, *p* = 0.01; reaction time: *χ*^2^ = 17.2, *p* < 0.001, df = 2).

### Imaging data

#### PET analysis

H3R availability was calculated as V_T_ for each ROI. V_T_ data were normally distributed (ACC: *p* = 0.49, DLPFC: *p* = 0.93; Shapiro-Wilk test of normality) with no outliers present. We found no statistically significant effect of group on V_T_ (ACC: control mean ± SD = 15.7 ± 2.7, patient mean ± SD = 14.2 ± 1.8, DLPFC: control = 11.2 ± 2.8, patient = 10.4 ± 1.4, main effect of group F_1,21_ = 0.79, *p* = 0.38; repeated measures ANOVA), or group-by-ROI interaction (F_1,21_ = 1.95, *p* = 0.18). We found no significant volumetric difference between groups in either the DLPFC (control = 36066 ± 5356, patient = 35085 ± 6075, t_22_ = 0.42, *p* = 0.68) or ACC (control = 40195 ± 4521, patient = 36819 ± 3131, t_22_ = 2.13, *p* = 0.05). As the volumetric difference in the ACC was trending towards significance, we included it as a covariate when comparing group differences of V_T_ and did not find a significant effect (F_2,21_ = 3.10, *p* = 0.07).

#### fMRI analysis

Working memory-related neural activation (defined as contrast of 1- & 2-back minus 0-back contrast) in both controls and patients can be seen in Fig. 2B and C respectively, with activation patterns seen in both groups, including the prefrontal and cingulate cortices. Figure 2D displays group differences in activation, yielded by a mixed-effects group-level analysis using FSL cluster thresholding (Z = 2.3, *p* < 0.05 cluster corrected for multiple comparisons), highlighting significantly higher activated clusters applying the above condition in controls compared to patients. We found significantly greater activation in controls compared to patients in the whole brain analysis, with local maxima being located within the right (MNI coordinates: x = 22, y = −30, z = −32) and left (x = 14, y = −36, z = −54) cerebellum. We did not find any brain regions that showed significantly greater working memory-related activation in patients compared to controls.

For ROI-specific analysis, parameter estimates were extracted from atlas-derived masks predefined during PET analysis using the 1- & 2-back > 0-back condition. Parameter estimates from the ACC were not normally distributed (*p* = 0.03), while those of the DLPFC were normally distributed (*p* = 0.75). No outliers were detected in either ROI. There was no significant difference in mean parameter estimates between groups, using the 1- & 2-back > 0-back condition, in the ACC (control mean ± SD = −10.2 ± 15.1, patient mean ± SD = −8.7 ± 20.0, U = 86.00, *p* = 0.44) or DLPFC (control = 12.4 ± 22.5, patient = 4.5 ± 27.3, t_22_ = 0.77, *p* = 0.45).

#### PET – task performance correlations

**Fig. 3:**
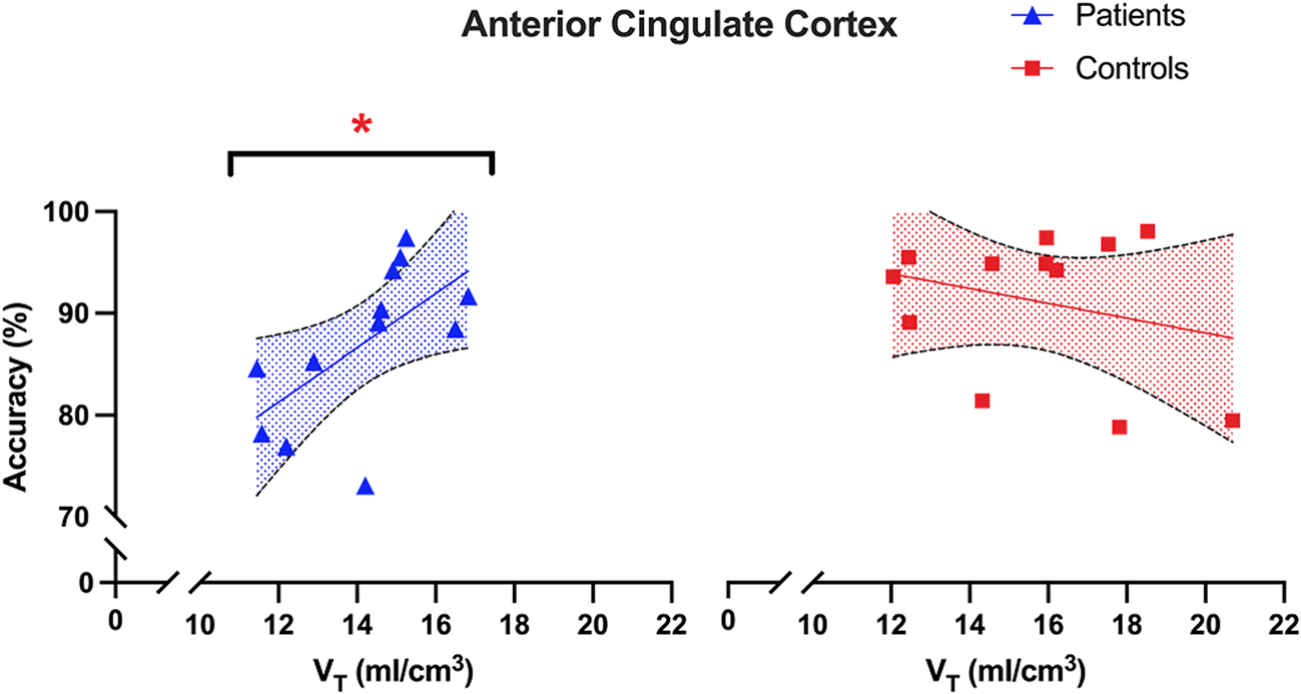
Relationship between task performance and H3R availability in the ACC. H3R availability measured as [^11^C]MK-8278 tracer uptake, and task performance measured as total task accuracy. H3R had a significant positive correlation with task performance in patients (rho = 0.73, *p* = 0.007; α = 0.05/4 = 0.0125) but not in controls (rho = 0.03, *p* = 0.94). V_T_, volume of distribution; *, α < 0.0125; shaded area, 95% confidence intervals

Correlation analyses were performed including task performance data, measured as total task accuracy pooled over task conditions, and PET data, namely ACC and DLPFC V_T_. In the ACC, we found a significant correlation between task performance and H3R availability in patients which survived correction for multiple comparisons (rho = 0.73, *p* = 0.007; α = 0.05/4 = 0.0125) (Fig. 3). Post-hoc analysis found significant correlation between H3R availability and performance for task conditions with higher working memory load (0-back: rho = 0.52, *p* = 0.08; 1-back: rho = 0.68, *p* = 0.02; 2-back: rho = 0.65, *p* = 0.02). This relationship was absent in controls (rho = 0.03, *p* = 0.94). We found a trend towards a significant difference in the correlation coefficients between patients and controls for the relationship between ACC V_T_ and working memory performance (r_diff_ = 0.70, z = 1.91, 95% CI = −0.03–0.95, *p* = 0.06). In the DLPFC, we found no significant correlation in either controls (Pearson’s *r* = −0.54, *p* = 0.09) or patients (*r* = 0.17, *p* = 0.63). Similarly, there was no significant difference between correlation coefficients for the DLPFC (r_diff_ = −0.71, z = −1.65, 95% CI = −0.94–0.15, *p* = 0.10).

#### PET – fMRI correlations

Turning to PET-fMRI correlations, we found no significant correlation between V_T_ and mean parameter estimate (beta values) data extracted from the ACC ROI, in either controls (rho = −0.07, *p* = 0.82) or patients (rho = −0.22, *p* = 0.49). Similarly, within the DLPFC, we found no significant correlation in controls (*r* = 0.22, *p* = 0.52) or patients (*r* = −0.21, *p* = 0.55). As there were no group differences in either PET or fMRI data, we ran an exploratory analysis grouping patients and controls together, to increase statistical power of the analysis to determine if a relationship existed between H3R and working memory-related activation in the ACC and DLPFC. We found no significant correlation in either the ACC (rho = −0.22, *p* = 0.30) or the DLPFC (*r* = 0.09, *p* = 0.71).

## Discussion

Our main finding is a strong positive correlation between ACC H3R availability and n-back task performance in patients with schizophrenia, which is absent in healthy volunteers. In the DLPFC, we found no significant relationship between H3R availability and task performance in either group. In contrast to our hypothesis, we did not find a significant relationship between H3R and working memory related task-evoked activation in the DLPFC or ACC in either group.

To our knowledge, this is the first reported finding that H3R availability is positively correlated with cognitive task performance in patients with schizophrenia. The n-back task engages sustained attentional memory during the 0-back condition, with higher working memory load applied sequentially in the 1-back and 2-back conditions (Owen et al. 2005). Interestingly, our findings are in contrast to preclinical data, which has indicated that both H3R knock-out (Rizk et al. 2004; Bizon et al. 2012) and antagonism (Wallace et al. 2011; Vohora and Bhowmik 2012) improves task performance, although these tasks (i.e., Barnes and Radial-arm maze) focussed on time taken to reduce errors rather than task accuracy. It is postulated that this is due to H3R antagonism increasing synaptic signalling, via increased synthesis and release of neurotransmitters (e.g., dopamine, glutamate, and acetylcholine) in cortical regions (Arrang et al. 1987; Schlicker et al. 1988, 1989, 1993; Clapham and Kilpatrick 1992; Morales-Figueroa et al. 2019). However, the primary function of H3R is as an autoreceptor, regulating the synthesis and release of histamine itself (Arrang et al. 1983). In vitro evidence has found that higher densities of H3R, in the absence of agonists or antagonists, are linked to increased constitutive activity and thus reduced histamine synthesis and release (Morisset et al. 2000; Moreno-Delgado et al. 2006). There is preclinical evidence that suggests a reduction of histamine transmission can improve the learning time and performance of working memory-analogous tasks (Klapdor et al. 1994). Lesions of the tuberomammillary nucleus, which is the histaminergic nucleus and the sole source of histamine pathways, have been found to facilitate learning of avoidance tasks in rodents (De Almeida and Izquierdo 1986; Segura-Torres et al. 1996). Moreover, one study reported that H3R agonism and reduction in cerebral histamine concentrations improved spatial memory task performance (Rubio et al. 2002). Taken together, these findings suggest the strong association seen in patients with schizophrenia between H3R availability and task performance, may be attributed to H3R reducing cerebral histamine transmission.

However, this relationship between H3R availability and task performance was not identified in our control group. Post-mortem data have found increased histaminergic turnover in the cerebrospinal fluid of patients with schizophrenia compared to controls, which suggests increased histamine activity in schizophrenia (Prell et al. 1995). The concentrations of histamine metabolites had a moderately positive correlation with positive symptom severity (*r* = 0.45). Through these findings, one could hypothesise that due to the higher activity of histamine in schizophrenia, namely via increased synthesis and release, H3Rs may have a more prominent function in autoinhibiting histamine release, and therefore have a more significant impact on working memory compared to healthy volunteers. However, further studies are necessary to assess this, by characterising the concentrations of histamine metabolites in the CSF of patients and examining its relationship with both in vivo H3R availability and working memory-task performance.

Our results found BOLD activation patterns during the n-back task for both patients and controls that have been reported in previous studies, including frontoparietal activation (Owen et al. 2005). ROI analysis did not find a significant difference in activation for either control > patient, or patient > control contrasts in the ACC or DLPFC. In part this may be due to a small sample size, which might not have been sensitive to detecting group differences at the chosen threshold. Whole-brain analysis found significantly lower activation in patients compared to controls within the cerebellum. This is consistent with meta-analytic data showing reduced activation within the cerebellum of patients with schizophrenia relative to controls in working memory tasks (Bernard and Mittal 2015). This is thought to be due to reduced forward modelling during working memory tasks, that includes utilising the experience to develop task intuition. This involves engagement of the cerebellum and cerebello-cortical loops which relieves prefrontal cortical processing that is typically activated during the early stages of working memory tasks (Ito 2008; Stoodley et al. 2012). However, despite identifying a relationship between H3R and task performance, we did not find a significant relationship between H3R availability and working memory-related BOLD activation extracted from topographically identical ROIs. A previous study quantifying H3R availability in healthy volunteers and investigating the association with fMRI based BOLD activation during the n-back, found a significant negative correlation in the right middle frontal gyrus (Ito et al. 2018). Methodological differences may have contributed to the conflicting results we found, including the use of an alternative H3R radioligand, image acquisition parameters, and the study in healthy volunteers employed resampling procedures through bootstrapped samples due to their report of a small sample size. Further studies with larger sample sizes would be of benefit to explicate the relationship between the DLPFC H3R availability and working memory task activation.

The association between H3R availability and working memory performance was restricted to the ACC and not found in the DLPFC. Notwithstanding this relationship with working memory performance, there was no association between H3R availability and working memory-related BOLD activation in the ACC. Working memory task performance studies in schizophrenia have found that the ACC is related to error commissioning, with fMRI evidence of increased activity in the ACC compared to hypoactivity in the DLPFC being associated with impaired attentional control and higher demands for error monitoring (Carter et al. 2001; Glahn et al. 2005). Additionally, although the n-back task is effectively employed as a task that engages working memory, a core facet of working memory is attention, with higher attentional-control demands noted in higher task conditions (Kane et al. 2007). Although working memory tasks and tasks specifically assessing attention have similar activation patterns, comprising parietal and occipital regions, key differences have been noted where there is higher activation in frontal regions with increased working memory-load but not attentional-load (Tomasi et al. 2007). These aspects of additional cognitive processes that are attributed to the ACC and can be engaged in working memory tasks, may contribute to our findings of a correlation with performance and H3R availability solely in the ACC of patients, without a relationship with DLPFC H3R availability. Moreover, in previously published work, we found an inverse relationship in healthy volunteers between H3R availability in the DLPFC and executive function task performance, which was absent in patients with schizophrenia (Arumuham et al. 2023). Future studies would be of benefit to investigate whether variable H3R regional expression affects distinct cognitive domains.

### Strengths and limitations

A strength of our study was the use of a selective and high affinity radioligand (inhibition constant: 0.54nM) for the quantification of H3R availability, with only weak off-target binding observed at 5-HT_2_ and 5-HT_2A_ receptors out of 170-receptors screened (Van Laere et al. 2014). Thus, [^11^C]MK-8278 is a selective tool for quantification of H3R availability, particularly in cortical regions where the average test-retest variability is 5.0 ± 4.1% including the ACC and DLPFC (Van Laere et al. 2014). Furthermore, to limit a temporal confounding effect of the diurnal variation of histamine release in the CNS (Mochizuki et al. 1992; Brown et al. 2001; Burns et al. 2003), all participants received scans during the same time of the day.

A limitation to consider is that our study was not powered to detect weak correlations. Thus the possibility of a Type II error should be considered for the lack of significant relationships with the fMRI data. These relationships warrant further investigation with larger cohorts to reduce the risk of both Type I and II errors, and thus stengthen conclusions. Although the majority of recruited patients were medication free (*n* = 7), the remaining participants were treated with either olanzapine or risperidone which have neglible affinity for H3R but a higher affinity for H1R (Appl et al. 2012; Kaar et al. 2020). Although this binding profile and thus antipsychotic exposure is unlikely to have a meaningful impact on our findings, we cannot completely exclude potential downstream changes to H3R expression secondary to chronic H1R antagonism. A limitation associated with the cross-sectional design employed for this study, is that it does not prove causality. Future studies should consider a drug challenge design utilising H3R modulating compounds, to specifically examine the impact of H3R modulation on working memory task performance to test causality.

### Implications of findings

The strong correlation between ACC H3R expression and n-back performance in patients, but not in controls, could be due to increased histaminergic turnover (Prell et al. 1995, 1996), with subsequent variation in H3R function. Interestingly, compared to other antipsychotics, clozapine has the highest affinity for H3R as an agonist (Kaar et al. 2020), and has been found to improve general cognition, including working memory, in patients with schizophrenia (Lee et al. 1994; McGurk 1999; Woodward et al. 2005). Despite not delineating the direction of causality, the sum of these findings could suggest higher constitutive activity and activation of H3R may improve working memory deficits seen in schizophrenia (Rubio et al. 2002; McCutcheon et al. 2023).

## Conclusions

Our findings provide evidence of a positive correlation between ACC H3R availability and cognitive task performance in patients with schizophrenia, which is absent in controls. We also found overall higher working memory task-evoked activation of cerebellar regions in controls relative to people with schizophrenia, in line with previous research (Bernard and Mittal 2015). We did not find evidence of a significant relationship between H3R availability and neural correlates of working memory load in the ACC or DLPFC of either group. Our results provide further evidence for the relationship between H3R and cognitive function. Further multi-modal imaging studies, including assessment of central histamine transmission, and implementing specific H3R modulating drugs would elucidate the role of H3R in cognitive processes.

## Supplementary information

Below is the link to the electronic supplementary material.

ESM 1 (DOCX 4.76 MB)

## Data Availability

The data that support the findings of this study are available from the corresponding authors, AA and ODH, upon reasonable request.
