# Supplementary material for: Working memory processes and the histamine-3 receptor in schizophrenia: a [11C]MK-8278 PET-fMRI study
Source: Psychopharmacology (Berl). 2024 Dec 22;242(6):1321–34. doi: 10.1007/s00213-024-06730-6 (PMC12084182; doi:10.1007/s00213-024-06730-6)
Supplement: Supplementary file 1 — (DOCX 4.76 MB) [file 213_2024_6730_MOESM1_ESM.docx]

**Supplementary material**

**Methods**

**Inclusion and exclusion criteria**

Inclusion criteria for all participants: 1) between 18­65 years of age; 2) capacity to consent to participation in the study; 3) modified Allen’s test showing adequate collateral circulation to the hand (to ensure safety of radial arterial catheterization); 4) no history of clotting or renal abnormality and no abnormal blood results on screening blood test; 5) English speaking sufficient to understand task instructions and information sheet; 6) in females, a negative urine pregnancy test at screening visit, and before PET and MRI scan. 7) for patients, a Diagnostic and Statistical Manual of Mental Disorders (DSM-IV) diagnosis of a schizophrenia according to the *Structured Clinical Interview of DSM-IV-TR Axis I Disorders-Patient Edition*.

Exclusion criteria for all participants: 1) ages <18 or >40; 2) a history of a head injury resulting in loss of consciousness; 3) personal history of serious medical illness; 4) contraindication to MRI scanning (e.g., metallic implants, claustrophobia, inability to lie comfortably for 90 minutes); 5) radiation exposure that would take total exposure (including participation in this study) to >10mSv in 12 months; 6) current or lifetime history of substance use or dependence as determined by the Structured Clinical Interview for DSM-IV-TR (SCID-I/P); 7) screened positive for any of the following substances (except cannabis) on a multi-panel urine drug screen detecting the following substance; amphetamine (300ng/ml cut off), cocaine (150 ng/ml cut off), ketamine (1000 ng/ml cut off), cannabis (50ng/ml cut off), methamphetamine (300 ng/ml cut off), opiates (2000 ng/ml cut off) (SureScreen Diagnostics, Derby); 8) current or recent use (no use within 3 months) of histaminergic drugs including, but not limited to, drugs with H3 affinity such as pitolisant, and other antihistaminergic drugs); 9) donation of blood or blood products in excess of 500ml within any 60 day period prior to the present study; 10) for healthy volunteers, a lifetime history of an Axis-I psychiatric disorder (DSM-IV) or confirmed diagnosis of Axis-I disorder in 1^st^ degree relatives.

**PET Image Analysis: Pre-processing Methods**

### *Data pre-processing*

Data pre-processing was performed using a combination of Statistical Parametric Mapping 12 (<http://www.fil.ion.ucl.ac.uk/spm>) and FSL (<http://www.fsl.fmrib.ox.ac.uk/fsl>) functions, as implemented in MIAKAT (<http://www.imanova.co.uk)>. Motion correction was applied for all PET scans. During motion correction, cumulative movement (CM) was defined as the sum of frame-by-frame Euclidean distance. As there was no correlation found between V_T_ estimates and CM in the ROIs considered, CM was not included in further statistical analysis.

Attenuated corrected frames were realigned to a single “reference” frame, by employing a mutual information algorithm. This created a movement-corrected dynamic image used in the analysis. Realigned frames were summated to create an individual motion-corrected reference map for brain tissue segmentation. Specifically, individual T1-weighted MR images were co-registered to the PET image using rigid body transformation. Normalisation parameters were obtained by warping the co-registered structural MRI to MNI space (International Consortium for Brain Mapping ICBM/MNI) using bias-corrected segmentation in SPM12. The inverse of these parameters was used to fit the Clinical Imaging Centre (CIC) atlas to each individual PET scan (Tziortzi et al., 2011).

*Blood data processing*

Blood input functions (both whole blood data and plasma data), POB and PPf modelling were performed using MultiBlood, a unified framework for the arterial data modelling to achieve an accurate and fully automated description of the plasma tracer kinetics (Tonietto et al., 2015). The pipeline employed pursuit techniques for estimating both radio-metabolites and parent concentration models from the raw plasma measurements, allowing the resulting algorithm to be both robust and flexible according to the quality of data available.

*Kinetic analysis*

For the ROI analysis, we implemented the standard two-tissue compartmental model (2TCM), expanding the previous analysis to describe [^11^C]MK-8278 brain kinetics (Van Laere et al., 2014). Identification of model parameters were done using nonlinear estimator (matlab lsqnonlin.m) and weighting each data point for the inverse of its error variance. The blood volume parameter (Vb) was fixed at 5% to reduce the error induced by the noisy blood input functions into the parameter estimates.

The anterior cingulate cortex (ACC) and dorsolateral prefrontal cortex (DLPFC) ROIS were obtained from the CIC neuroanatomical atlas (Tziortzi et al., 2011). Using Statistical Parametric Mapping 12 (SPM12; version 6684)(The FIL Methods Group, 2014) grey matter (GM) masks were obtained by binarising segmented GM from T1-weighted images and applying this to the CIC atlas.

*Quality control*

The outputs of the image analysis were manually controlled for by experienced PET modelers. Specifically we tested that 1) brain extraction did not include loss of brain or excess of non-brain tissue, 2) GM, white matter (WM) and cerebrospinal fluid (CSF) were properly extracted, 3) both MNI structural template and CIC atlas were aligned to individual MRI, 4) PET frames were realigned to the same space correcting for subject inter-frame motion, 5) the fitting of the blood input function was physiological (e.g., no negative values), and 6) the fitting of brain PET data provided by kinetic modelling was physiological (i.e., 2TCM). Scans failing any of point 1 to 6 were labelled as having failed quality control and excluded from analysis.

*Kinetic Modelling Validation*

A 2TCM model demonstrated good fit of the data, both in patients and controls (see s-Figure 1). Compared to a one-tissue compartmental model (1TCM), 2TCM provided superior fitting performance and lower Akaike information index estimates for 77% of the cases analysed (all ROIs and all subjects). V_T_ estimates from Logan graphical analysis demonstrated to be significantly correlated with 2TCM-based V_T_ estimates in all ROIs.

**s-Figure 1: Time Activity Curves for a representative control and patient, including 1TCM and 2TCM**


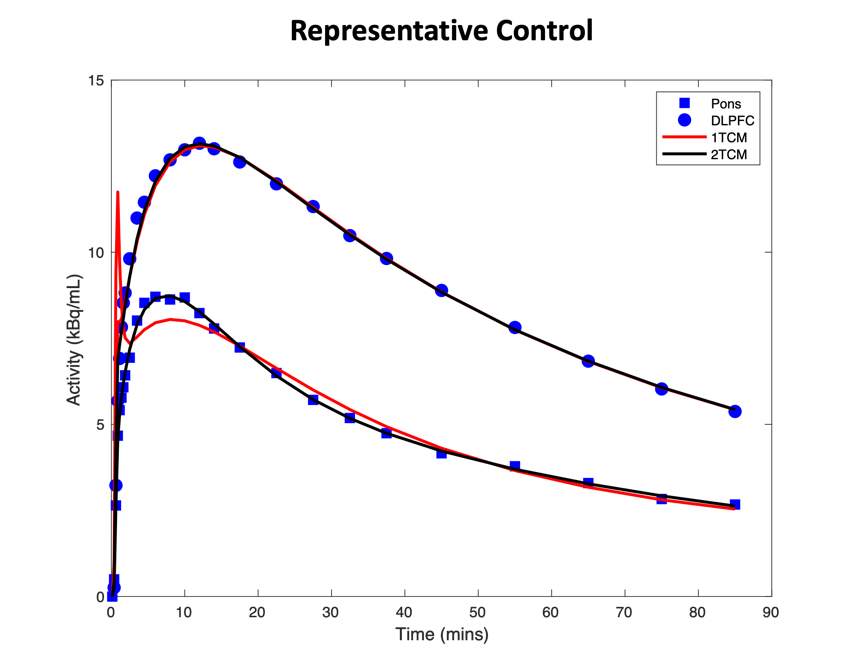

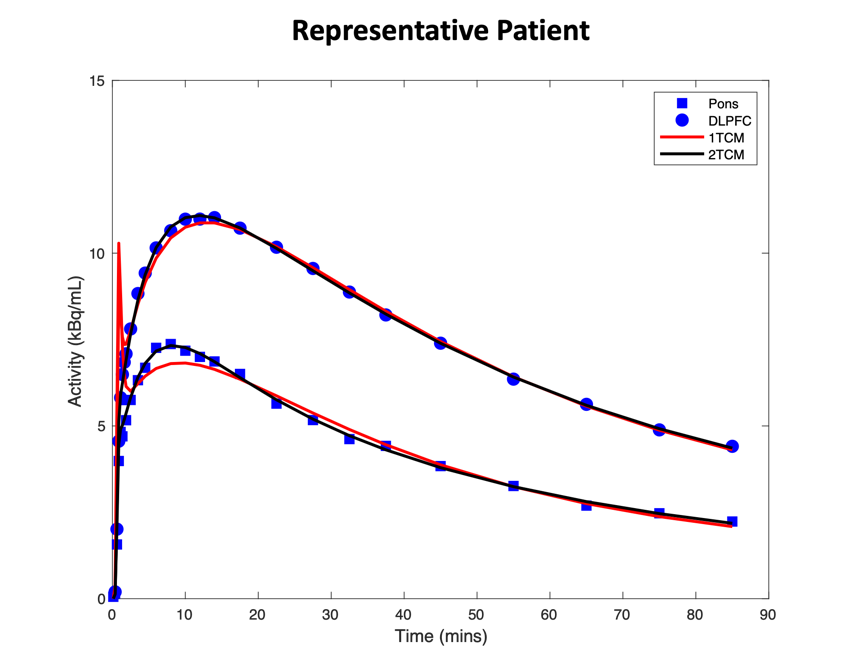


Regions indicated in these graphs include the dorsolateral prefrontal cortex (DLPFC) and pons, which were an example region and the region of lowest volume of distribution, respectively. Modelling indicated that 2TCM had a greater fit for the current data compared to 1TCM.

**s-Figure 2: DLPFC PET and task performance correlations**

**
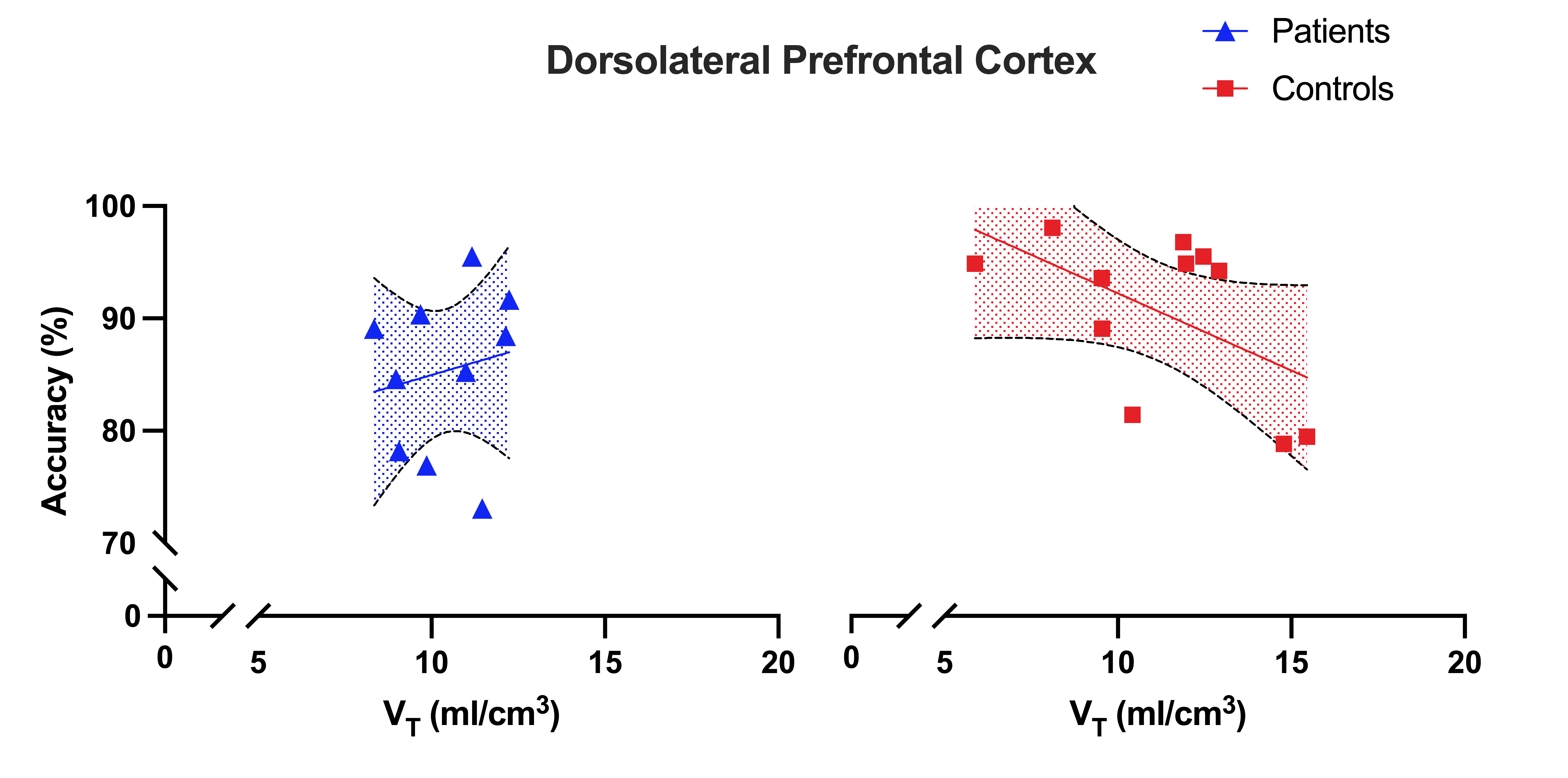
**

Graphical representation of the relationship between [^11^C]MK-8278 tracer uptake and total (0-, 1-, & 2-back conditions) task performance, determined by accuracy of response, in the DLPFC. The shaded areas indicate 95% confidence intervals.

**s-Figure 3: ROIs PET and fMRI BOLD activation correlations**

**

**

Graphical representation of the relationship between [^11^C]MK-8278 tracer uptake and task-evoked BOLD activation, measured as parameter estimates for the contrast 1- & 2-back > 0-back. S-3A and S3-B indicate data from the ACC and DLPFC respectively. The horizontal dashed line in the figure represents y = 0. The shaded areas indicate 95% confidence intervals.

**s-Figure 4: Masks developed for ROIs data extraction**


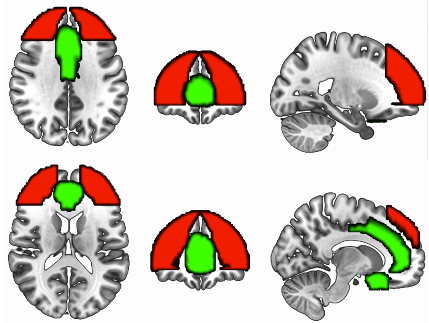


Masks derived for regions of interest (ROIs) data extraction for both anterior cingulate cortex (ACC) (highlighted in green) and dorsolateral prefrontal cortex (DLPFC) (highlighted in red), using the Clinical Imaging Centre (CIC) neuroanatomical atlas. Masks were applied to both PET and fMRI data, thereby providing anatomically identical ROIs for each data set.

**s-References:**

The FIL Methods Group, 2014. Statistical Parametric Mapping Software, Wellcome Trust Centre for Neuroimaging. Institute of Neurology, University College London.

Tonietto, M., Veronese, M., Rizzo, G., Zanotti-Fregonara, P., Lohith, T.G., Fujita, M., Zoghbi, S.S., Bertoldo, A., 2015. Improved models for plasma radiometabolite correction and their impact on kinetic quantification in PET studies. J. Cereb. Blood Flow Metab. 35, 1462–9. https://doi.org/10.1038/jcbfm.2015.61

Tziortzi, A.C., Searle, G.E., Tzimopoulou, S., Salinas, C., Beaver, J.D., Jenkinson, M., Laruelle, M., Rabiner, E.A., Gunn, R.N., 2011. Imaging dopamine receptors in humans with [11C]-(+)-PHNO: dissection of D3 signal and anatomy. Neuroimage 54, 264–277. https://doi.org/10.1016/j.neuroimage.2010.06.044

Van Laere, K.J., Sanabria-Bohorquez, S.M., Mozley, D.P., Burns, D.H., Hamill, T.G., Van Hecken, A., De Lepeleire, I., Koole, M., Bormans, G., de Hoon, J., Depre, M., Cerchio, K., Plalcza, J., Han, L.L., Renger, J., Hargreaves, R.J., Iannone, R., 2014. 11C-MK-8278 PET as a Tool for Pharmacodynamic Brain Occupancy of Histamine 3 Receptor Inverse Agonists. J. Nucl. Med. 55, 65–72. https://doi.org/10.2967/jnumed.113.122515
